# Advances in the Structural and Physiological Functions of SHARPIN

**DOI:** 10.3389/fimmu.2022.858505

**Published:** 2022-04-25

**Authors:** Beiming Yu, Feng Wang, Yanfeng Wang

**Affiliations:** Key Laboratory of Molecular Medicine and Biotherapy, School of Life Science, Beijing Institute of Technology, Beijing, China

**Keywords:** SHARPIN in LUBAC, SHARPIN alone, structure, cellular role, physiological function

## Abstract

SHARPIN was initially found as a SHANK-associated protein. SHARPIN can be used as an important component to form the linear ubiquitin chain assembly complex (LUBAC) with HOIL-1L, HOIP to produce a linear ubiquitin chain connected N-terminal Met1, playing a critical role in various cellular processes including NF-κB signaling, inflammation, embryogenesis and apoptosis. SHARPIN alone can also participate in many critical physiological activities and cause various disorders such as chronic dermatitis, tumor, and Alzheimer’s disease. Mice with spontaneous autosomal recessive mutations in the SHARPIN protein mainly exhibit chronic dermatitis and immunodeficiency with elevated IgM. Additionally, SHARPIN alone also plays a key role in various cellular events, such as B cells activation and platelet aggregation. Structural studies of the SHARPIN or LUBAC have been reported continuously, advancing our understanding of it at the molecular level. However, the full-length structure of the SHARPIN or LUBAC was lagging, and the molecular mechanism underlying these physiological processes is also unclear. Herein, we summarized the currently resolved structure of SHARPIN as well as the emerging physiological role of SHARPIN alone or in LUBAC. Further structural and functional study of SHARPIN will provide insight into the role and underlying mechanism of SHARPIN in disease, as well as its potential application in therapeutic.

## 1 Introduction

Proteins are biomolecules that perform life activities. Proteins in cells are in the process of continuous synthesis, modification and degradation, and the level and quality of intracellular proteins are closely related to the normal function of the cells (1). According to the current study, The two main protein degradation pathways in the body are autophagy lysosome system (AL) pathway and ubiquitin proteasome system (UPS) pathway (2). The ubiquitin proteasome system (UPS) pathway is a tightly regulated pathway targeting degradation with specific proteins. Proteins that are degraded by the ubiquitin proteasome system (UPS) pathway are usually abnormal proteins, as well as short-lived proteins. In this way, the cellular activity can be strictly regulated (3–6).

Ubiquitin(Ub), is a small-molecule globular protein widely found in eukaryotic cells (5). The ubiquitination process involved three enzymes: a Ub-activating enzyme (E1), a Ub-conjugating enzyme (E2), and a Ub ligase (E3) (1). Ub itself can be ubiquitinated to form eight different types of isoform poly-Ub chains, including K6, K11, K27, K29, K33, K48, K63 as well as M1 homopolyubiquitin, heteroubiquitin as well as branched ubiquitin chains (1). These different types of ubiquitin, also known as the “ubiquitin code”, have different effects on the life activities of proteins and cells (6). Different types of ubiquitin chains regulate various substrates, playing important role in the biological event of cells. The K6-linked ubiquitin chain is involved in the autophagy process of mitochondria as well as phagocytosis (7). The K11-linked ubiquitin chain has been identified as one of the proteasomal degradation signals for cell cycle regulation (8). Related studies have found that the ubiquitin chains linked to K27 are involved in DNA damage repair (9). The K29-linked ubiquitin chain is a proteasomal degradation signal (10). The ubiquitin chain linked by K33 is involved in the transport of post-Golgi membrane proteins (11). The K48-linked ubiquitin chain is the most common chain type in proteasome degradation. However, the linked ubiquitin chain of K63 was shown to be involved in DNA repair and signaling, but not acting as a degradation signal (12). As a unique type of poly-Ub chain, the linear poly-Ub chain (also called the M1-linked poly-Ub chain) is widely involved in both innate and adaptive immune signaling pathways (13). Each ubiquitin molecule contains, besides Lys, serine, threonine, and tyrosine residues that can alter the structural characteristics of the ubiquitin chain through modifications such as phosphorylation, acetylation, and methylation.

The LUBAC is the only E3 ligase complex that catalyzes the synthesis of linear ubiquitin chains (14). SHARPIN is an important component to form the LUBAC with HOIL-1L, HOIP to produce a linear ubiquitin chain connected N-terminal Met1, playing a critical role in various molecular and cellular processes including NF-κB signaling, inflammation, embryogenesis and apoptosis (13, 15, 16). In addition, it was also found that HOIL-1L not only catalyzed the formation of oxygen-ester bonds between the C-terminal carboxyl group of ubiquitin, but also modulated immune signaling and cell death *via* monoubiquitination of LUBAC (17, 18). Some studies have identified the HOIP homolog, LUBEL, in drosophila. The LUBEL is mainly involved in the stress response of the Drosophila organism (19). Linear ubiquitination is unique to animals, and precisely because of its specificity, the discovery of linear ubiquitin chains and LUBAC is considered a pattern shift in the field of ubiquitin research. The absence of any of these three LUBAC subunits leads to immune-related diseases (20, 21).

SHARPIN is a protein composed of 387 amino acids, was originally identified as a SHANK binding protein (22, 23). SHARPIN is widely expressed in different cell types and tissues (24). For example, SHARPIN in rats is relatively abundant expressed in multiple organs and is mainly localized in the cytoplasm (25, 26). Some of the SHARPIN protein can also be found in the cell membrane folds and cell nucleus (27). Mice with spontaneous autosomal recessive mutations in SHARPIN predominantly exhibit chronic dermatitis and immunodeficiency of elevated IgM, called chronic proliferative dermatitis mice (cpdm) (28, 29). SHARPIN is not only involved in the inflammatory response but also suppresses integrin activation and plays a key role in tumor cells and macrophages that cause Alzheimer’s disease. The diversification of the SHARPIN function is inseparable from its particular molecular structure. SHARPIN contains three domains: PH, UBL, and NZF. The PH domain at the N-terminal is implicated in integrin and tumor regulation (30). The NZF at the C-terminal can bind ubiquitin, and the UBL domain is involved in forming the LUBAC (31). The full-length and NZF structures have not yet been resolved.

In this review, we summarize the now resolved structures of SHARPIN and its located LUBAC structure, introducing the cellular and physiological functions of LUBAC involvement and SHARPIN alone, including in immune diseases, tumors and other related diseases. Further structure and function study of SHARPIN genes and proteins will help to elucidate the pathogenesis of some tumors and some immune deficiency.

## 2 Structural Study of SHARPIN

### 2.1 SHARPIN Is Structurally and Functionally an Important Component of LUBAC

**Figure 1:**
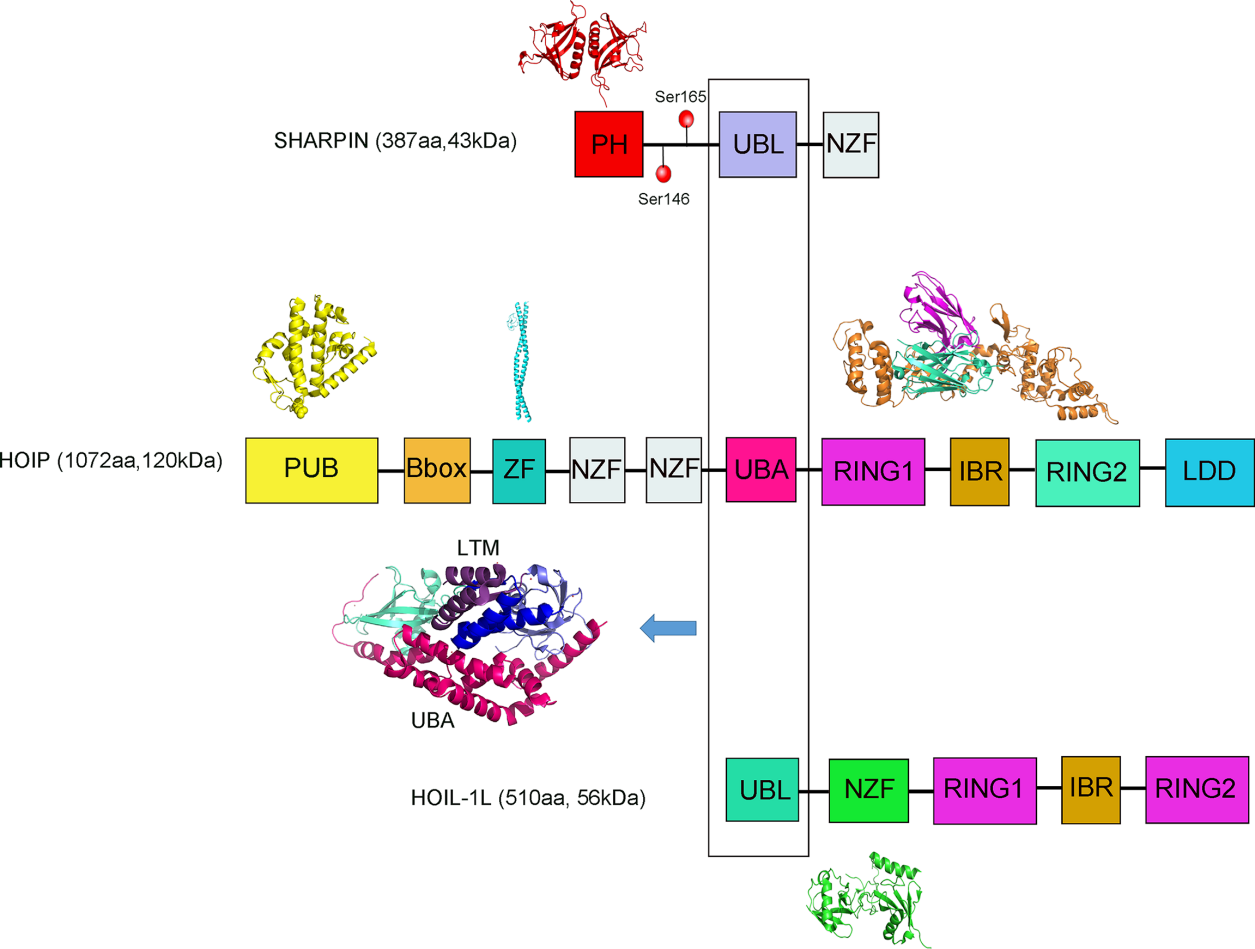
A schematic diagram showing the domain architectures of SHARPIN, HOIP, and HOIL-1L proteins. Structures are shown where available and include the PH domain of SHARPIN (4EMO); HOIP PUB (4OYK), HOIP ZF/NEMO UBAN (4OWF), HOIL-1L UBL/HOIP UBA/SHARPIN UBL complex(5Y3T), HOIP RBR/UBE2D2-ubiquitin (5EDV) and the HOIL-1L NZF/linear diUb complex (3B08).The phosphorylation of Ser165 of SHARPIN can be involved in the activation of NF-κB. The phosphorylation of Ser146 of SHARPIN promotes the metastatic spread of cancer cells through an interaction with ARP2/3.

SHARPIN contains the UBL domain (ubiquitin-like domain, UBL) and the NZF domain (Npl4-zinc finger domain, NZF), where, UBL recognizes the UBA domain (ubiquitin-associated domain UBA) of HOIP of LUBAC (**Figure 1**) (32). HOIP contains mainly a PUB domain (UBX-containing protein, PUB), a B-box ZF, a canonical ZF, two NZF domains, a UBA domain and C-terminal the RBR domain (RING-between RING, RBR), coupled with the HOIP-specific extension of the domain LDD (linear Ub chain determining domain, LDD) (32). HOIP interacts with E2 with ubiquitin through its RING1 structure and transfers ubiquitin from E2 to a specific Cys residue at position 885 (Cys885) on the RING2 domain. Subsequently, a peptide bond is catalyzed on the Cys885 between the carboxyl group of the beta-amino group of the receptor ubiquitin, eventually recognized by LDD. HOIL-1L also contains the UBL domain and the NZF domain (32). Both HO1L-1L and HOIP contain RBR domains and are members of the RBR ubiquitin ligase E3 family, the RBR domain contains two RING domains to which RING1 can bind E2, has the properties of RING class E3, and RING2 can form thioester intermediates with ubiquitin. The similarity in HOIL-1L is also achieved *via* NZF binding to ubiquitin. The NZF mutation in HOIL-1L blocked the binding of linear ubiquitin chains and exacerbated the inflammatory response in cpdm mice but did not interfere with the activity of LUBAC (33). The HOIP alone is in an autoinhibited state and autoinhibition of HOIP is released upon complex formation with HOIL-1L and SHARPIN (**Figure 1**) (31).

Although SHARPIN is unable to produce linearized ubiquitin modifications, they can activate LUBAC activity when they bind to HOIP. The LUBAC plays an absolutely key role in conjugating the linear ubiquitin chain to the substrates such as NEMO (**Table 1**). At the same time, NEMO has a specific ubiquitin-binding region that interacts with LUBAC (34, 35). NEMO deficiency leads to reduce the role of LUBAC in NF-κB signaling pathway and prevents SHARPIN-mediated linear ubiquitination, consistent with SHARPIN deficiency leads to inhibition of LUBAC-mediated linear poly-ubiquitination of endogenous NEMO (36, 37).

**Table 1:** SHARPIN connected with pathophysiological processes *via* different signaling pathway.

Targeted Process	Diseases	Cellular effects	Targeted signaling pathway	SHARPIN Overexpression/Knockdown
Immune response	Chronic proliferative dermatitis mutation (cpdm) mice	Cpdm mice are deficient in SHARPIN and develop dermatitis, multi-organ pathology and an immunological phenotype	SHARPIN deficiency blunts the TNFR1 pro-survival transcriptional signal and sensitizes cells to TNF-induced cell death	SHARPIN deficiency can sensitize TNF/TNFR1-induced caspase-8-mediated apoptosis and thus hyperapoptosis induces an inflammatory response
	Branchio-oto-renal (BOR)	Characterized by kidney defects, hearing loss, and branchial arch abnormalities	SHARPIN can enhance the function of the Eya protein	The BOR syndrome was demonstrated in zebrafish after knockdown of SHARPIN
	Inflammatory NF-κB signaling pathway	SHARPIN-mediated linear ubiquitination regulates the NF-kB signaling activation	Promote IkBα deubiquitinatin	P50 and p65 levels were elevated in activated NF-κB complexes after LUBAC overexpression
	Platelet aggregation	SHARPIN inhibits integrin activation and platelet aggregation	SHARPIN is found to be expression in platelets, can respond associated with aIIbβ3, inhibit aIIbβ3 activation with aIIb	Loss of SHARPIN correlates with increased β3-intergin activity
Cancer	Tumorigenesis	SHARPIN expressing CHO-K7 cells produced 1.6 times more colonies than the vector-transfected cells	SHARPIN suppresses the PIP3 (phosphorylation of 3 phosphate, PIP3) phosphorylation through its ubiquitin-like domain and PTEN interaction	The tumorigenic properties of CHO-K7 cells are significantly promoted upon SHARPIN overexpression, suggesting that SHARPIN has proto-oncogenic effects
		SHARPIN promotes the formation of lamellipodium through the ARP2/3 complex	SHARPIN serine 146 phosphorylation promotes the metastatic spread of cancer cells through an interaction with ARP2/3	Overexpressed SHARPIN promotes cancer cell proliferation, tumor formation and cancer metastasis
	Breast cancer	Regulates the ERαprotein ubiquitination and promote breast cancer growth	Overexpression of SHARPIN significantly increased ERα protein levels	Overexpression of SHARPIN in breast cancer is correlated with ERα protein levels, and that SHARPIN can promote breast cancer development by regulating the ubiquitination of ERα proteins, during which SHARPIN is not involved in regulating gene expression
		Depleted SHARPIN resulted in decreased cell proliferation and enhanced expression of p53	SHARPIN modulates the p53 protein levels through poly-ubiquitination and degradation in a MDM2-dependent manner	Knock down SHARPIN enhanced expression of p53
	Colon carcinoma cell	SHARPIN with PRMT5 contributes to regulating the transcription of cancer-associated genes	SHARPIN regulates p53 through PRMT5-dependent signaling with PAX3 and MITF	Unknown
	Esophageal cancer	SHARPIN inhibits Esophageal cancer cell progression by promoting YAP degradation	SHARPIN can promote YAP K48-linked ubiquitination and degradation	Modulation SHARPIN expression level could inhibit cancer cell progression in Esophageal cancer
	Renal cell carcinoma	SHARPIN promotes the development of renal cell carcinoma	Enhance pVHL protein ubiquitination and degradation	Overexpression of SHARPIN leads to elevated intracellular HIF2α in patients with Renal cell carcinoma. SHARPIN can promote polyubiquitination of pVHL and thus degradation by the proteasome, which causes HIF-2α to escape the fate of ubiquitination and successfully into the nucleus to function
	Melanoma	SHARPIN promotes Melanoma Progression	SHARPIN upregulates Rap activation which can promote the invasion and metastasis of human melanoma cells	Unknown
Neurodegenerative diseases	Alzheimer’s disease (AD)	SHARPIN-mediated inflammation in macrophages exposed to Aββ promoted the neuronal cell death	SHARPIN is required for NLRP3 activation	SHARPIN knockdown attenuated Aβ-induced NLRP3 expression in macrophages

### 2.2 Structure of SHARPIN in the Ternary LUBAC

The stability of the LUBAC is determined by the interaction between the three subunits. It has been shown that HOIP interacts with the UBA domain of both the HOIL-1L and SHARPIN subunits, through its UBL domain. The cryogenic electron microscopy structure of the HOIP UBA domain in ternary complex with HOIL-1L UBL domain and SHARPIN UBL domain was resolved at an overall resolution of 2.4 Å (**Figure 1**) (31). A low-resolution three-dimensional map of full-length LUBAC was obtained by negative staining electron microscopy of the recombinant complex (38). The results showed a dispersed distribution of similar size particles under cryoEM, and its 2D classification showed a distinct elongated dumbbell structure. At the same time, a 3D reconstruction of the obtained particles was performed, but due to the too low resolution, only the overall structure of the LUBAC presents an elongated asymmetric crescent-shaped outline, and most of the mass is concentrated in one (38). They propose HOIP and HOIL-1L can act synergically to produce branches of heteromorphic ubiquitin chains containing linear and non-K-linked chains of oxygen-linked branches (38). Among these three interactions, they are interactions by HOIP UBA with SHARPIN UBL and HOIP UBA with HOIL-1L (31). In addition, the ternary complex structure shows that the newly discovered LTM motifs (LUBAC-tethering motif, LTM) between the HOIL-1L and SHARPIN subunits co-folds into a spherical domain, this unique interaction-formed structure plays an key role in maintaining the stability of LUBAC. Subsequently, the heterodimerization of the LTMs stably formed the TD (Tethering domain, TD) (31, 39).

HOIL-1L and SHARPIN form a spherical structure by LTMs heterodimerization, promoting the stabilization of the two UBLs on the HOIP UBA, thus contributing to the maintenance of the ternary LUBAC core conformation (31). Furthermore, loss of either HOIL-1L or SHARPIN will profoundly destabilize LUBAC. However, the outcome of such a loss differs depending on the subunit: loss of HOIL- 1L causes embryonic lethality, whereas loss of SHARPIN develops severe auto-inflammatory disease and immunodeficiency in mice (**Table 1**) (29, 40). This discrepancy may be due to a difference in the ability of SHARPIN and HOIL-1L to stabilize HOIP. LUBAC stabilization *via* the LTM-mediated interaction is a general mechanism (31, 41).

### 2.3 Structural Study of SHARPIN Alone

SHARPIN consists of the PH domain, UBL domain, and NZF domain. The PH domain employs a highly conserved pleckstrin homology super folding and is commonly used as a scaffold for building protein interaction modules. The PH domain cannot function as a ligand recognition domain to recognize and bind ubiquitin, because it lacks many of the surface properties present in other interaction modules based on pleckstrin homologous folding (30, 42). By parsing the crystal structure, a tetrameric structure of PH was obtained, with a resolution of 2.0 Å (**Figure 1**) (30).

Val-114 is located at the center of the hydrophobic interface formed by the other half of the dimer. Single electrostatic charges incorporated by the center of the interface hydrophobic frame eliminated the interactions by mutant Val-114 to Asp (30).

The current structure suggests that the PH domain is thought to be unrelated to the catalytic activity of LUBAC and that it may play a role in other physiological functions, such as its tumor-related role or inhibition of integrin activation (23). The structure of PH and UBL domain has been resolved in the current structural analysis of SHARPIN, but the structure of full-length SHARPIN and NZF domain is unknown, and the mechanism of how PH is involved in tumor regulation and the mechanism of SHARPIN in binding to ubiquitin are also unclear.

## 3 The Cellular Roles of SHARPIN

### 3.1 SHARPIN-Mediated Linear Ubiquitination Regulate the NF-κB Signaling Activation

**Figure 2:**
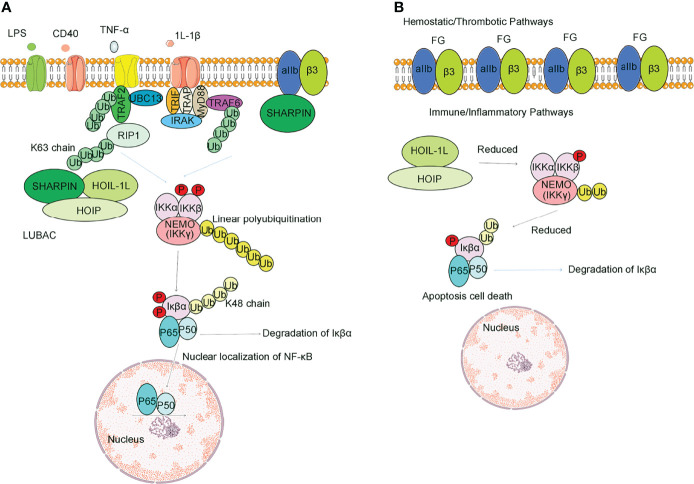
Difference between SHARPIN presence and absence in regulating the NF-κB signaling pathway and integrin activation. **(A)** Normal SHARPIN expression. SHARPIN is found to be expression in platelets, can respond associated with aIIbβ3, inhibit aIIbβ3 activation with aIIb, and can also participate in the formation of LUBAC, thereby promoting Met1 ubiquitination. Stimulation of platelets through receptors for TNFα, LPS, 1l-1β or CD40 activates LUBAC to add Met1 linear ubiquitin chains (yellow circles) to NEMO, provoking transautophosphorylation of IKKβ and phosphorylation of IκBα. In nucleated cells, Lys-48-ubiquitination (yellow circles) results in degradation of IκBα, which frees NF-κB for nuclear translocation. **(B)** Effects of SHARPIN knockdown. Reduction of SHARPIN levels associated with αIIb prime αIIbβ3 activation and fibrinogen binding. Reduction in SHARPIN levels also destabilize LUBAC. Only a selected number of factors of the TNFR1-associated signaling complex are depicted.

Mammalian NF-κB signaling is an inducible family of transcription factors with central roles in multiple aspects of cellular homeostasis, including cell proliferation, survival and apoptosis, development of lymphoid tissues and mobilization of intrinsic immune and acquired immune responses (43, 44). The NF-κB signaling pathways are typically divided into two classical and non-classical pathways based on the properties of the signaling inhibitory components (**Figure 2**) (45). Classical pathways are principally triggered by TNF, LPS, T cells and B cell receptors, acting as the primary NF-κB pathway in most cells. The classical NF-κB signaling pathway includes the classical IκB complex, which mainly consists of IKKα, IKKβ and the subunit NEMO (a regulator necessary for the NF-κB pathway, also known as IKKγ) (46, 47). TNF-α and 1L-1β, two inflammatory cytokines, are the earliest and most important inflammatory mediators during the inflammatory response (48, 49). Following TNF-α activation, the K63 strand is involved in NF-κB activation, and TAK1-TAB1-TAB2 is a key component. K63 polyubiquitination of RIP1 induced by TNF-α has been hypothesized to be a key part of recruiting NEMO and TAK1, where IKKα and IKKβ phosphorylate and activate IKK, and TAK1 is thought to act as the upstream kinase to phosphorylate IKKs. Through the NZF1 domain of HOIP, the LUBAC recognizes its substrate NEMO and linearly ubiquitinates NEMO. Linear-ubiquitinated NEMO has a higher affinity for recognizing IKKα and IKKβ, thus activating the IKKs. Then, activated IKKs would phosphorylate IKβα. Phosphorylated IKβα can expose P50 and P65, allowing P50 and P65 into the nucleus to activate NF-κB (50). Some scholars demonstrated that NEMO was recruited because it contains a UBAN domain, which has a high affinity for both the K63 and linear ubiquitin chains (16, 51, 52). Non-classical pathways are mediated mainly through members of the tumor necrosis factor receptor superfamily, such as the B cell activation factor BAF, lymphotoxin LT and CD40 ligands (53).

The Rel family proteins of p50 and p65 levels were elevated in activated NF-κB complexes after LUBAC overexpression (**Figure 2**) (54). Knockdown of the LUBAC reduces the basal or TNF-α-activated NF-κB activity (55). LUBAC is an upstream regulator of the IKK. Further studies revealed that binding of the coiled-leucine zipper domain of NEMO to the ZF domain of HOIP led to the linear ubiquitination of NEMO mediated by LUBAC, and substitution of arginine for these lysines inhibited the ubiquitin linearization of NEMO and LUBAC binding and inflammatory factor-mediated activation of the NF-κB pathway (43). It has been reported that the phosphorylation of Ser165 of SHARPIN can be involved in the activation of NF-κB. If the serine at position 165 is mutated, the LUBAC can still form, but the NF-κB transcriptional activity is reduced, and delayed nuclear translocation of p65 and p50 occurs and leads to reduced binding to DNA (56).

DNA toxic agents, such as antitumor agents, can lead to the activation of the NF-kB signaling pathway (57). The DNA damage response first stimulates the ataxia-telangiectasia gene (ataxia telangiectasia mutated) kinase that belongs to the phosphatidylinositide 3-kinase. Activation of ATM (ataxia telangiectasia mutated, ATM) induced phosphorylation of Lys277 and Lys309 of NEMO. Phosphorylated NEMO can inhibit protein c-IAP mono-ubiquitination, further prompting the formation of IKβα and activating NF-κB signaling pathway (58). It was shown that NF-κB pathway activation is required under genotoxic effects and that LUBAC linearly ubiquitylates NEMO with intracellular modifications and further activates TAK1 and IKK. Therefore, it is suggested that LUBAC-mediated linear ubiquitination modification of NEMO under genotoxic stress protects cells from apoptosis due to DNA damage (59, 60).

Through the *in vivo* experiments, knock out of the HOIL-1L gene in HEK293T cells, it can be observed that SHARPIN-HOIP still induces the NF-κB transcriptional activity. However, another study showed that NF-κB activation is reduced and delayed in HOIL-1L KO cells (42, 61). Moreover, SHARPIN-HOIP exhibited a linear polyubiquitination activity *in vitro* polyubiquitination assay (42). In addition, resection of SHARPIN reduces CD40 activity and further leads to CD40 recruitment to IKK in the B cell (39). It is thus suggested that the linear ubiquitination activity of SHARPIN is essential for the signaling regulation of CD40. Deletion of SHARPIN substantially reduces the number of LUBAC (62). Furthermore, LPS is a membrane complex on gram-negative bacteria that can cause septic shock in the body. The effect of LPS-mediated NF-κB pathway activation was attenuated in SHARPIN null mutant cpdm mice, suggesting a role of LUBAC in the intrinsic immune role of activation and a greatly reduced IL-1β production in macrophages in cpdm mice with Toll-like receptor ligands, also implicating SHARPIN in Toll-like receptor signaling (63).

### 3.2 SHARPIN Can Limit Cell Death

LUBAC is essential for embryogenesis because it can prevent cell death and promote hematopoiesis. Once mice lack SHARPIN or HOIP, they can lead to severe dermatitis or embryonic lethality, respectively (64). Inflammation can cause programmed cell death, including apoptosis, necrosis, pyroptosis and so on (63). Necrosis is uncontrolled: cells explode and release their contents into their surroundings (63). Inflammation is activated by a protein called normal tumor necrosis factor (TNF) receptor 1 (TNFR1). SHARPIN plays a key role in the TNF signaling pathway (65). Mutations in SHARPIN can increase the sensitivity of keratinocytes to TNF-induced caspase-8-mediated apoptosis (66). This implies that a key role of SHARPIN is to limit cell death.

### 3.3 SHARPIN Promotes B Cell Activation

CD40 is essential for antibody isotype conversion, antibody production, and humoral immune memory production. Degradation of kBα inhibitor (IkBα) mediated by CD40 leads to activation of NF-κB (67). It was found that LUBAC is abundant in B cells and T cells. When organisms are immune-stimulated, LUBAC is recruited to the CD40 receptor signaling complex, whereas the degree of NF-κB pathway activation resulting from this process is attenuated in SHARPIN mutant cpdm mice (28). Knockdown of HOIP reduced the activity of CD40 and abolished the recruitment of the IKK in the B cell line (68). It was considered that the linear ubiquitination activity of LUBAC is essential for the regulation of CD40 signaling in B cell (68).

### 3.4 SHARPIN Inhibits Integrin Activation and Platelet Aggregation

Integrins are heterodimers of α and β subunits (69). Common integrins include α1β1, α2β2 and αIIbβ3. Integrins deploy their affinity for the ligands through allosteric regulation (70). The regulation of integrin activity is critical in the development of cell-cell attachment, supporting cell anchoring, stabilizing organization and organismal structure (71, 72). Some researchers thought SHARPIN is an inhibitor of integrin activity (73). SHARPIN inactivates integrins platelets and megakaryocyte cells and influences integrin-dependent cell-cell attachment. SHARPIN silencing enhanced the cell surface activity of β1-integrins (73). In addition, deletion of SHARPIN promoted increasing β1-integrin activity *in vivo (* 74, 75).

Platelets are specialized hemostatic and immune cells (76). Integrin αIIbβ3 is required for platelet aggregation (77). SHARPIN is found expressed in platelets, can respond associated with aIIbβ3, inhibit aIIbβ3 activation with aIIb, and can also participate in the formation of LUBAC, thereby promoting Met1 ubiquitination (78). In the normal state of SHARPIN expression, SHARPIN associates with αIIbβ3 (79, 80). Stimulation of platelets through receptors for thrombin, LPS, or sCD40L activates LUBAC, provoking transautophosphorylation of IKKβand phosphorylation of IKβα (**Figure 2**) (77).

**Figure 3:**
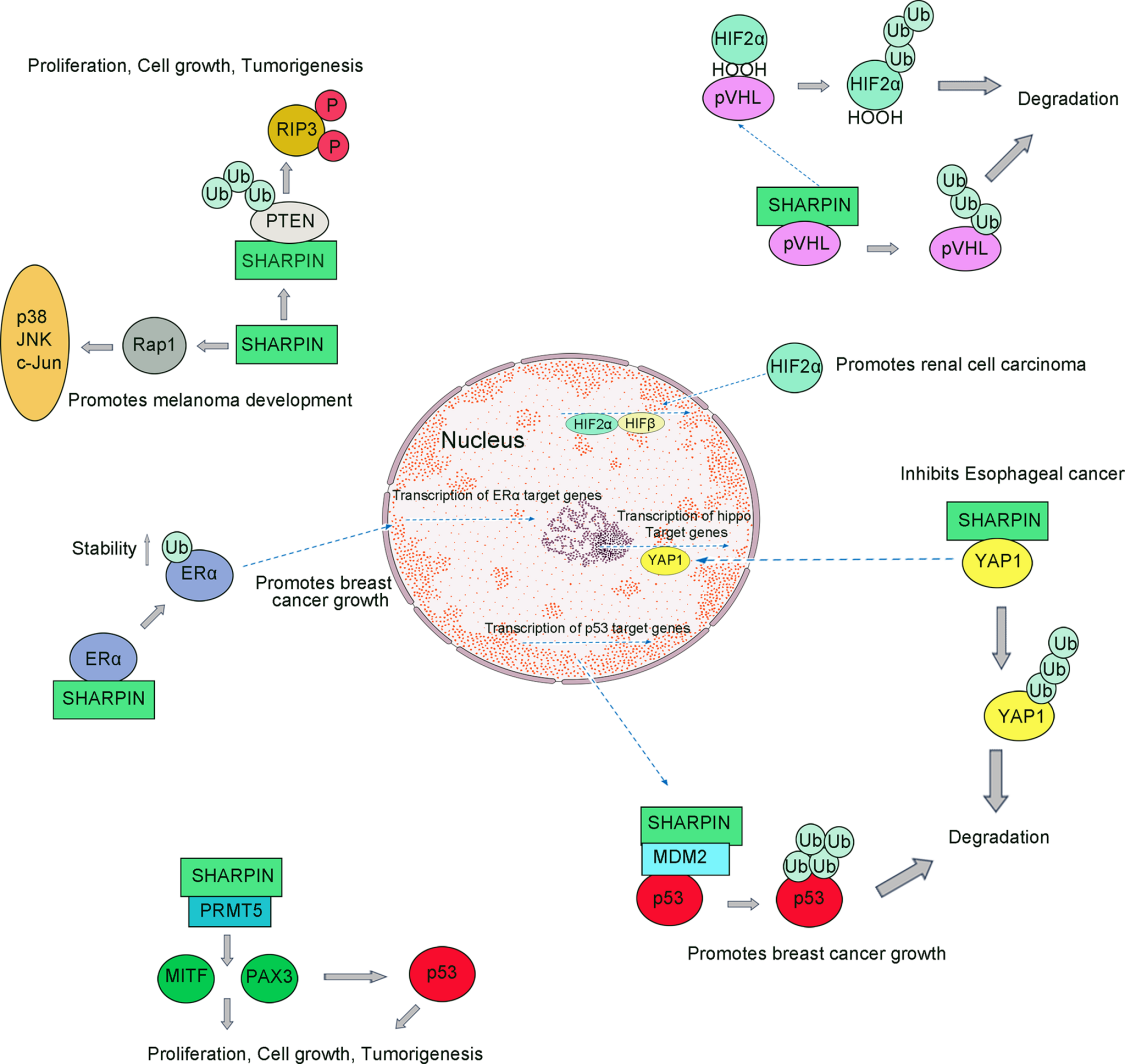
SHARPIN regulates multiple signaling pathways during tumorigenesis. SHARPIN suppresses the PIP3 phosphorylation through its ubiquitin-like domain and PTEN interaction which mediates tumorigenesis; SHARPIN upregulates Rap activation which can promote the invasion and metastasis of human melanoma cells; SHARPIN can promote breast cancer development by regulating the ubiquitination of ERα proteins; SHARPIN regulates p53 through PRMT5-dependent signaling with PAX3 and MITF, which promotes tumorigenesis; SHARPIN also functions to inhibit P53 protein stability *via* MDM2; SHARPIN inhibits Esophageal cancer cell progression by promoting YAP K48-linked ubiquitination and degradation; SHARPIN can promote polyubiquitination of pVHL and thus degradation by the proteasome, which causes HIF-2α to escape the fate of ubiquitination and successfully into the nucleus to function which promotes the development of renal cell carcinoma.

In anucleate platelets, activation of the intracellular LUBAC components can induce the occurrence of nongenomic responses (81, 82). SHARPIN knockdown results in reduced levels of αIIb binding and initiates αIIbβ3 fibrinogen binding. Reduction in SHARPIN levels decreases intracellular LUBAC stability and activity. A dramatic decrease in the megakaryocyte/platelet lineage promotes the function of platelets in immunity and inflammation (83, 84). Moreover, SHARPIN can regulate integrin inactivation by interacting with Rap1 (Ras-associated protein-1, Rap1) in cells (**Figure 3**) (85). Rap1 activation can promote the invasion and metastasis of human melanoma cells (86).

### 3.5 SHARPIN Role in Bone Formation

Cpdm mice exhibited low cortical bone density, reduced volume and quantity of trabecular, indicating that osteogenesis was impaired in cpdm mice (87). Eya1(Eyes absent 1 protein, Eya1) was important in the development of bone and kidney in both invertebrates and vertebrates (**Table 1**) (88). Mutations in the Eya1 gene are linked to BOR (Branchio-oto-renal, BOR) syndrome, characterized by kidney defects, hearing loss, and branchial arch anomalies. SHARPIN was found to be an important interaction partner of Eya1. SHARPIN can enhance the function of Eya1 proteins. The BOR syndrome was demonstrated in zebrafish after the knockdown of SHARPIN, implying SHARPIN plays a role in bone and craniofacial formation (88–90).

## 4 The Pathophysiological Functions of SHARPIN

### 4.1 SHARPIN and Chronic Dermatitis

Chronic proliferative dermatitis mutation (cpdm) mice lack SHARPIN and develop dermatitis (91, 92). Auto-inflammatory diseases are caused by defects or dysregulation of the innate immune system. The inhibition of linear ubiquitination leads to the production of autoinflammatory diseases and immunodeficiency diseases (65). Because the only cause of cell death in the absence of a SHARPIN is the abnormally increased TNF signaling, which suggests that TNF/TNFR1-mediated cell death is responsible for the cpdm phenotype (93). Moreover, SHARPIN deficiency can also sensitize caspase-8-mediated apoptosis and thus hyperapoptosis induces an inflammatory response (94).

### 4.2 SHARPIN and Tumor

Normal human SHARPIN protein was initially found to locate in the cytoplasm, but employing immunohistochemistry in ovarian and hepatocellular carcinoma found SHARPIN expressed in both the cytoplasm and the nucleus (95). Alternatively, overexpression of SHARPIN in Chinese hamster ovarian cells can promote cell migration and cell proliferation (25). At present, several gene expression analyses targeting tumor biopsy tissue are underway, and the human SHARPIN gene is upregulated in tumorigenesis in various human cancers such as breast cancer, esophageal cancer and renal cell carcinoma (**Table 1**) (96–98).

#### 4.2.1 SHARPIN Promotes Tumorigenesis by Promoting PTEN Inactivation

First, SHARPIN mutant mice exhibiting chronic proliferative dermatitis suggest that SHARPIN is not only an indolent scaffold protein but may also activate cell migration and proliferation, and therefore may be involved in the aberrant proliferation of tumor cells. Furthermore, PTEN (phosphatase and tensin homolog, PTEN) as a tumor suppressor gene, its inactivation is associated with tumorigenesis (99). Current evidence suggests that the PTEN-NRs (negative regulatory protein of PTEN, PTEN-NRs) plays a role in the inactivation of PTEN at tumorigenesis, and SHARPIN is a PTEN-NR (100).

In human primary cervical cancer cells, SHARPIN suppresses the PIP3 (phosphorylation of 3 phosphates, PIP3) phosphorylation through its ubiquitin-like domain and PTEN interaction (**Figure 3**) (101). Aberrant SHARPIN expression promotes growth mediated by xenografted tumor cells in immunodeficient mice (102). SHARPIN expression is associated with the loss of PTEN function. Taken together, it is suggested that SHARPIN is a PTEN-NR and that it promotes the tumor by inhibiting the function of PTEN, and at least in part promotes tumorigenesis.

Some scientists have established stable CHO-K7 cells after transfection of the SHARPIN expression vector, which shows excessive SHARPIN expression compared to normal human cells (25). Under equivalent conditions without serum, cells expressing SHARPIN grew faster than in the control group. SHARPIN expressing CHO-K7 cells produced 1.6 times more colonies than the vector-transfected cells (25). Given that cell mobility and invasiveness are key features of the malignant cancer cells, the effect of SHARPIN on cell mobility was investigated by a wound-healing assay. The wound closure rate of cells expressing SHARPIN was significantly faster compared to controls after 5h, as determined by the Matrigel invasion assay. SHARPIN has proto-oncogenic effects and may be involved in cancer development and progression (100, 103).

#### 4.2.2 SHARPIN Serine 146 Phosphorylation Promotes the Metastatic Spread of Cancer Cells Through an Interaction With ARP2/3

The leading cause of cancer-related death is the metastatic spread of cancer cells. The ARP2/3 complex is responsible for creating branched actin networks. Overexpression of the ARP2/3 complex is closely associated with tumor cell invasion (104). SHARPIN promotes the formation of lamellipodium through the ARP2/3 complex (105). The ARP2/3 complex can be used as a marker to differentiate benign lesions from malignant melanoma (106). The phosphorylation on SHARPIN at serine 146 selectively mediates SHARPIN’s interaction with the ARP2/3 complex, a protein interaction that may provide a target for therapeutic interference in cancer (105).

#### 4.2.3 SHARPIN Promotes Breast Cancer by Increasing the Stability of ERα Signaling

Breast cancer is the highest diagnosed cancer in females worldwide (96, 107). Some studies have shown that the abnormal function of transcription factors/nuclear receptors such as ERα (estrogen receptor alpha, ERα) promotes the development of breast cancer (108, 109). Molecular biological studies had shown that SHARPIN can promote breast cancer development by regulating the ubiquitination of ERα proteins, during which SHARPIN is not involved in regulating gene expression (110). Recent studies have shown that SHARPIN stabilizes ERα by promoting mono-ubiquitination of ERα at K302/303 residues and that this stability can be disrupted by mutation at K302/303 residues (**Figure 3**) (111, 112). In addition to its involvement in breast cancer progression, SHARPIN is also strongly associated with metastasis in breast cancer.

#### 4.2.4 SHARPIN-Mediated Regulation of Tumor Cell Metastasis *via* Inhibiting p53 Signaling

In most cancer cells, p53 exhibits functional inactivation (113). Some studies have shown that activated p53 can be involved in many downstream responses and can regulate many important physiological processes, including DNA repair, apoptosis, and tumorigenesis (114). In normal cells, p53 binds with the E3 ubiquitin ligase MDM2 (mouse double minute 2 homolog, MDM2) and exhibits very low cellular levels, which contributes to the ubiquitination of p53 and hence its rapid degradation by the proteasome (**Figure 3**) (34).

A recent study indicated that SHARPIN may be upstream of p53 signaling in breast cancer cells because the depleted SHARPIN leads to reduced cell proliferation and enhanced p53 expression (115). Furthermore, a study reported that SHARPIN regulates p53 protein levels through MDM2-dependent polyubiquitylation. Thus, SHARPIN can indirectly regulate p53 protein levels in cells (115).

PRMT5 is a member of the PRMT family (116). It is reported that PRMT5-knockdown destabilize p53 (117, 118). Several studies have confirmed that PRMT5 is essential for p53 protein synthesis and that in colon cancer cells (119). Studies have also shown that SHARPIN can interact with PRMT5, which can facilitate the transcription of regulatory cancer-related genes (120, 121). SHARPIN can be involved in the regulation of PRMT5 activity, in turn increasing the proportion of transcriptional activity of PAX3 and MITF involved in melanoma growth (122).

#### 4.2.5 SHARPIN Inhibits Esophageal Cancer Cell Progression by Promoting YAP Degradation

Esophageal cancer is one of the most common malignant tumors, accounting for a relatively large number of newly diagnosed cases in China, or about 60% (123). Cancer causes include known environmental factors including alcohol and smoking, and genetic factors (124). Based on genome sequencing analysis and molecular biological evidence indicated that the dysregulation of Hippo signaling is common in Esophageal cancer and that inhibition of YAP, a core factor of Hippo signaling, leads to reduced proliferation and invasiveness of esophageal cancer cells (125). Hippo signaling can control tissue growth and organ size, which is done accomplished by balancing the relationship between cell proliferation and cell death (126). The core hippo pathway consists of a kinase cascade: the upstream kinase MST1/2 phosphorylates and activates the downstream kinase LATS1/2. Activation of LATS1/2 can lead to the phosphorylation of the activator YAP. Elevated levels of phosphorylated YAP expression can cause the proliferation of Esophageal cancer cells (127).

A higher expression level of YAP was observed in esophageal cancer. Abnormally elevated levels of YAP protein promote tumor metastasis and affect later tumor stages. SHARPIN can promote K48-linked ubiquitination and degradation, and it can associate with YAP and promotes YAP degradation which in turn leads to reduced YAP transcriptional activity and the ability of cancer cells to progress (**Figure 3**) (97). On this basis, targeted inhibition of SHARPIN expression and activity may be a strategy for the treatment of esophageal cancer (**Table 1**).

#### 4.2.6 SHARPIN Promotes the Development of Renal Cell Carcinoma by Enhancing pVHL Protein Ubiquitination and Degradation

Renal cell carcinoma, originating from the renal tubular epithelial cells (128). About 17% of renal cancers were found to be associated with distant metastases (129). Hypoxia-related pathways are associated with the formation and progression of Renal cell carcinoma. pVHL (Von Hippel-Lindau protein, pVHL) is a tumor suppressor (130). In renal cell carcinoma, loss of function occurs in pVHL, leading to sustained activation of HIF (hypoxia inducer factor, HIF) signaling, controlling hypoxia-induced tumor growth and development by regulating the expression of relevant genes. In normoxia, HIF-2α is hydroxylated by a PHD (prolyl hydroxylase domain-containing protein, PHD), which is recognized by pVHL and then finally ubiquitinated and degraded by the proteasome (131).

Overexpression of SHARPIN leads to elevated intracellular HIF2α in patients with Renal cell carcinoma. SHARPIN can promote polyubiquitination of pVHL and thus degradation by the proteasome, which causes HIF-2α to escape the fate of ubiquitination and successfully transfer into the nucleus to control gene expression (98). Therefore, SHARPIN promotes the development of renal cell carcinoma *via* enhancing the degradation of the tumor suppressor pVHL.

### 4.3 SHARPIN and Alzheimer’s Disease

Alzheimer’s disease (AD) mainly includes the accumulation of hyperphosphorylated tau proteins and amyloid-beta(Aβ) in the brain (132, 133). During normal physiology, homeostasis is maintained between Aβ production and degradation, mainly influenced by immune cells (134). In some conditions, the main reason is related to senescence, and these cells fail to engulf Aβ. Excess Aβ accumulation is accompanied by reduced degradation, leading to chronic inflammatory activation of macrophages, microglia and thus the progression of AD.

A recent study found that the onset of late Alzheimer’s disease (LOAD) may be associated with functional variants in SHARPIN (132). Some studies showed that the expression of SHARPIN was significantly increased in the presence of Aβ, indicating a link between Aβexposure and SHARPIN expression in macrophages. NLRP3 inflammasome can mediate macrophage polarization. Here, SHARPIN gene knockdown attenuated NLRP3 expression in Aβ-induced macrophages (**Table 1**). A study found that it resulted in a loss of function of the SHARPIN, which is required for NLRP3 activation (135).

## Conclusion and Perspective

After a lot of research, great achievements have been made in studying the physiological role of SHARPIN. SHARPIN can not only mediate the activation of NF-κB signaling through linear ubiquitination regulation, but also regulate B cell activation and bone formation. SHARPIN can also inhibit integrin activity and is closely associated with both tumor and Alzheimer’s disease. At the molecular level, the study of SHARPIN structure is still in the preliminary stage. The partial protein structure of the SHARPIN protein has been resolved, including the crystal structure of PH domain and the UBL domain. However, the full-length structure of SHARPIN and LUBAC is not yet. Only part of the mechanism of SHARPIN is understood at the physiological level, most of the specific mechanisms remain unclarified. In particular, the relationship between SHARPIN and tumor development, tumor conversion will continue to become a research hotspot for a long time in the future. Further research into the function of SHARPIN genes and proteins will help to elucidate the pathogenesis of certain tumors, expected to be a new target for treating tumors.

## Author Contributions

YW and BY conceptualized, wrote the manuscript, made and revised the figures and tables. FW and YW discussed the paper. All authors contributed to the article and approved the submitted version.

## Funding

This work was supported by the National Natural Science Foundation of China Grants 32101021, the Youth Project of Beijing Natural Science Foundation 5214027, the National Natural Science Foundation of China Grants 31770827 and 21736002, the Beijing Institute of Technology Research Fund Program for Young Scholars.

## Conflict of Interest

The authors declare that the research was conducted in the absence of any commercial or financial relationships that could be construed as a potential conflict of interest.

## Publisher’s Note

All claims expressed in this article are solely those of the authors and do not necessarily represent those of their affiliated organizations, or those of the publisher, the editors and the reviewers. Any product that may be evaluated in this article, or claim that may be made by its manufacturer, is not guaranteed or endorsed by the publisher.

## Glossary

**Table:**

Ub	Ubiquitin
E1	Ub-activating enzyme
E2	Ub-conjugating enzyme
E3	Ub ligase
LUBAC	The linear ubiquitin chain assembly complex
SHARPIN	SHANK-associated RH domain-interacting protein
HOIP	Ring finger protein 31
HOIL-1L	RanBP-type and C3HC4-type zinc finger containing 1
cpdm	chronic proliferative dermatitis mice
UBL	ubiquitin-like domain
NZF	Npl4-zinc finger domain
UBA	ubiquitin-associated domain
RBR	RING-between RING
PUB	UBX-containing protein
ZF	zinc finger
NZF	Nlp4-like ZF domains
LDD	linear Ub chain determining domain
NEMO	Inhibitor of kappaB kinase gamma
NF-κB	Nuclear factor kappa B
LTM	LUBAC-tethering motif
TD	Tethering domain
ATM	ataxia telangiectasia mutated
IKKα	Nuclear factor of kappa light polypeptide gene enhancer, alpha
IKKβ	Nuclear factor of kappa light polypeptide gene enhancer,beta
Eya1	Eyes absent 1 protein
BOR	Branchio-oto-renal
TNF	tumor necrosis factor
TRADD	TNFR1-associated death domain protein
TRAF2	TNF receptor-associated factor 2
cIAPs	cellular inhibitor of apoptosis
RIPK1	receptor interacting protein kinase 1
PTEN	phosphatase and tensin homolog
PTEN-NRs	negative regulatory protein of PTEN
SIPL1	class SHANK interacting protein 1
PIP3	phosphorylation of 3 phosphate
ERα	estrogen receptor alpha
MDM2	(mouse double minute 2 homolog
Rap1	Ras-associated protein-1
Pvhl	Von Hippel-Lindau protein
HIF	hypoxia-induced factor
PHD	prolyl hydroxylase domain-containing protein
AD	Alzheimer’s disease
Aβ	amyloid-beta
LOAD	late-onset Alzheimer’s disease
